# Clinical Characteristics and Outcomes of COVID-19 in Children in Northern Iran

**DOI:** 10.1155/2021/5558287

**Published:** 2021-04-28

**Authors:** Leila Shahbaznejad, Hamed Rouhanizadeh, Mohammad Reza Navaeifar, Fatemeh Hosseinzadeh, Faeze Sadat Movahedi, Mohammad Sadegh Rezai

**Affiliations:** ^1^Pediatric Infectious Diseases Research Center, Communicable Diseases Institute, Mazandaran University of Medical Sciences, Sari, Iran; ^2^Student Research Committee, Mazandaran University of Medical Sciences, Sari, Iran

## Abstract

**Objective:**

Since December 2019, the coronavirus disease 2019 (COVID-19) has been spread rapidly all over the world, infecting all age groups with this novel virus. In this manuscript, we report characteristics of children with COVID-19 in Mazandaran province, northern Iran.

**Method:**

From 12 February to 28 July 2020, medical records of 100 children diagnosed with COVID-19 admitted to the hospitals of Mazandaran province were collected. Patients' age, gender, clinical symptoms, and signs, in addition to therapeutic management and outcomes, were reported.

**Results:**

57 (57%) boys and 43 girls with the mean age of 104.63 ± 79.14 months were evaluated. 20 patients (20%) were transferred to the PICU (pediatric intensive care unit), and 13 children experienced a severe form of the disease, pediatric inflammatory multisystem syndrome (PIMS). The mean duration of hospitalization was 5.3 ± 4.7 days. Fever (81%), respiratory (79%), gastrointestinal (47%), and neurologic complaints (29%) were experienced by the patients in addition to skin rash (14%). Sixty-two patients needed supplemental oxygen, and 6 of them underwent endotracheal intubation. Leukopenia was reported in 7, anemia in 24, and thrombocytopenia in 12 patients. 4 patients with underlying diseases including chronic renal failure, Down syndrome with cerebral palsy, and morbid obesity died.

**Conclusion:**

COVID-19 can cause symptoms in children in two stages. In the first week, upper and lower respiratory symptoms can occur which has lower severity and prevalence compared to adults. But after 2-3 weeks following infection, symptoms of MIS-C or multisystem involvement can occur and COVID-19 should be considered. The most common indication for admission is fever, rash, and respiratory problems.

## 1. Introduction

Since December 2019, the severe acute respiratory syndrome coronavirus 2 (SARS-CoV-2) has been spread rapidly around the world and also all age groups were infected with this novel virus [1]. In the first reports of coronavirus disease 2019 (COVID-19), the frequency of disease in children was lower than adults. In a study in China, until January 29, 2020, less than 1% of all patients were younger than 14 years [2]. Later on, when the disease spread to other countries and become well known in Korea, till 19 July 2020, 1.7% of total cases were under 9 years and 5.5% aged between 10 and 19 years [3]. The mortality rate in this age group was very low in both reports [2, 3].

Clinical manifestations of COVID-19 are rare or absent in children and adolescents [4]. The COVID-19 symptoms seem to be less severe in children than in adults [5]. The clinical presentation of pediatric patients may differ from those of the adults and can range from asymptomatic to acute upper respiratory tract infection, gastrointestinal symptoms with shock, or coagulation dysfunction in severe cases [6]. The most common complaint of children is usually nonspecific symptoms of upper respiratory tract infection, such as mild to moderate fever and cough [5]. Fever, rash, and shock with concomitant COVID-19 infection in children were called pediatric inflammatory multisystem syndrome (PIMS) associated with SARS-CoV-2 by the Royal College of Pediatrics and Child Health [7]. Similar to adults, children with comorbidities including chronic kidney and lung diseases, malignancies, diabetes, obesity, anemia, immune disorders, heart disease, and congenital malformations are more likely to develop severe conditions from COVID-19 [8]. In the study by Önal et al. [9] from Turkey, cough, fever, and weakness were reported as the most common complaints, and the majority of patients had mild to moderate signs of illness.

There is a lot of ambiguity in COVID-19 differences between adults and children regarding clinical symptoms, complications, and management. The regional or time variability in virus behavior is also unknown. Hence, early clinical detection of COVID-19 is essential to prevent further spreading [4]. This study is aimed at describing the characteristics and clinical manifestations of children with COVID-19 admitted to hospitals of Mazandaran province, north of Iran, as one of the first provinces involved during the first wave of the pandemic, and investigating prevalence of clinical symptoms, laboratory and radiological findings, and clinical outcomes. We also aimed to identify factors associated with pediatric COVID-19 infection. As the disease is novel and experiences about this virus are low, further understanding and reports of symptoms, clinical findings, and laboratory abnormalities in children may share valuable information with other practitioners and can better inform the ongoing efforts to control this global pandemic.

## 2. Methodology

### 2.1. Study Design and Interventions

We identified pediatric patients 1 day to 18 years of age with confirmed or suspected SARS-CoV-2 infection from 12 February 2020 to 28 July 2020 (5 months) admitted to 21 hospitals of Mazandaran province, northern Iran.

A COVID-19 case was defined as SARS-CoV-2 infection by a positive reverse transcription-polymerase chain reaction (RT-PCR) test of a specimen using a nasopharyngeal swab or positive serology (confirmed case), presence of clinical signs or symptoms and a COVID-19 compatible chest CT (computed tomography) scan (probable case), and clinical symptoms and history of known sick contact (suspected case).

Data from each patients' medical record were obtained through a research form. Demographic data including age, gender, and clinical signs and symptoms including fever, chills, cough, dyspnea, rash, nausea, vomiting, and diarrhea with no other reason were recorded [10, 11]. Chest CT involvement compatible with COVID-19 included patchy infiltration, ground-glass opacity, halo sign, reverse halo sign, and pleural effusion [12].

Clinical data including symptoms and signs in addition to laboratory data and radiologic results, therapeutic management, outcome, and mortality were also reported. Data were extracted from hospital records, hospital information system (HIS) software, and in some instances, telephone contact with parents of the children.

### 2.2. Outcomes

Outcomes reported included the need for invasive mechanical ventilation, hospital length of stay, and mortality during admission.

### 2.3. Statistical Analyses

Demographic and clinical characteristics were summarized as frequencies and percentages for categorical variables. Data were analyzed by using SPSS software, version 20.0. Written informed consent was obtained from parents of all patients.

## 3. Results

### 3.1. Demographic Characteristics

From a total of 130 COVID-19 hospital records referred for evaluation, 30 cases were excluded due to duplicate admission, inadequate data, or misdiagnosis with COVID-19, and finally, 100 children with the mean age of 104.63 ± 79.14 months (range: 1 day to 18 years) were studied. Of them, 57 were male and 70 children lived in the urban region.  Table 1 shows the demographic characteristics of the patients in detail. COVID-19 RT-PCR was performed for 54 patients, and 17 cases were positive (confirmed cases). So, the remaining 83 patients were probable or suspected cases. Twenty children were transferred to the PICU (pediatric intensive care unit) during hospitalization, and 13 of them experienced a severe form of the disease: PIMS. The mean duration of hospitalization in this study was 5.3 ± 4.7 (range: 1-37) days.

### 3.2. Clinical Symptoms

Fever (77%), cough (62%), and dyspnea (47%) were the most common chief complaints, and 14% of the patients had comorbid diseases.

During the disease course, 81 patients were febrile, and respiratory, gastrointestinal, and neurologic complications occurred in 79%, 47%, and 29%, respectively. Also, 14 children suffered from skin rash. The median duration of symptoms prior to admission was 5.53 ± 3.97 days.  Table 1 shows the clinical complaints of the patients in detail.

### 3.3. Patients' Medication

All of the patients received antibiotics; ceftriaxone (44%), vancomycin (33%), meropenem (25%), and azithromycin (19%) were the most commonly prescribed antibiotics. Eleven children received intravenous immunoglobulin (IVIG), 13 children received packed cell, and 9 children received albumin. Other treatment modalities like hydroxychloroquine and Kaletra were used for 44 and 19 patients, respectively (Table 2).

### 3.4. Lab Tests

Leukopenia was reported in 7, anemia in 24, and thrombocytopenia in 12 patients (Table 2). Laboratory data and symptoms of the children with COVID-19 infection are shown in Tables 3 and 4.

### 3.5. Underlying Diseases

Among 4 died patients, 2 children had chronic renal failure, another one was a Down syndrome with cerebral palsy, and the last one suffered from morbid obesity (16 years, weight 95 kg), and all of them underwent mechanical ventilation. Notably, all of these mortalities occurred in the first 6 weeks of the study period.

### 3.6. Ventilator Support

Sixty-two patients needed oxygen supplementary performed with noninvasive (mask, nasal cannula, and oxyhood) or invasive devices, of which 6 of them underwent endotracheal intubation, and just one of them survived.

## 4. Discussion

Fewer children than adults have been affected by the COVID-19 pandemic, and children have different clinical manifestations compared to adults [13]. The prognosis for COVID-19 pneumonia is good in children with no underlying diseases [14]. In this study, during 5 months, A total of 13983 patients were admitted to the hospitals of the Mazandaran province, of which there were 100 cases aged under 18 years (0.7%). Similar to us, in the United States, 5% of total infected patients were children but less than 1% of admitted cases were children [15]. In a study in China, 5.44% of the patients were younger than 16 years [16]. This difference in results may be due to different admission and outpatient policies in other countries.

Although the disease is rare in the neonatal period [1, 10, 11], 3% of admitted children in our study were newborns and 34% of the patients were under 5 years. In the study of Dong et al., 40% of children were aged under 5 years [17]. Qiu et al. and Shekerdemian et al. reported 28% and 30% of the children to be younger than 5 years, respectively [16, 18].

Like other studies [10, 12, 14, 19, 20], the most common presentations of our patients were fever and respiratory complaints including cough and tachypnea and all patients improved with an excellent prognosis. Gastrointestinal complaints like nausea and vomiting, abdominal pain, and diarrhea were also reported in some studies [12, 19, 20]. Neurologic complaints involved a significant number of children in this study, in a spectrum of drowsiness, confusion, headache, and convulsion. Neurologic complications were reported in pediatric COVID-19 [10, 12]. Fever, cough, and dyspnea were the most common presenting symptoms in Derespina et al.'s study [21]. Chao et al. reported cough and fever as the most common symptoms at admission [22]. In Dong et al.'s study, 731 cases of COVID-19 were confirmed in children, of whom more than 90% were asymptomatic or with mild to moderate symptoms [17].

In this study, the median duration of symptoms prior to admission was 5.53 ± 3.97 days. In Chao et al.'s study, patients reported a median duration of symptoms of 3 days before admission [22]. The median duration of symptoms prior to hospitalization was 5 days, and a known sick contact was reported in 50.8% of patients in Derespina et al.'s study [21]. The difference in duration of symptoms may be attributed to different hospitalization policies.

In our report, skin rash presented in 14% of the children. There are reports of dermatologic manifestation in COVID-19, and fever and rash were the first presentation of COVID-19 [12, 23, 24]. In some cases, the rash was associated with PIMS [25, 26].

Elevated acute phase reactants (ESR, CRP) were common in this study; although leukopenia, anemia, and thrombocytopenia were seen in a considerable number of the patients, these abnormalities were also reported in other studies [12, 14, 20, 26].

All of the patients in this study received antibiotics, mostly broad spectrum ones. As the SARS-coV2 is an invasive and novel virus, and there are many coinfection with other organisms, antibiotic prescription is very difficult because there is a concern about the rational usage of antibiotics and drug resistance [10]. Oseltamivir, hydroxychloroquine, and Kaletra were used as antivirals in this study. Although other researchers treated COVID-19 patients with antibiotics [12, 14, 20], there is no strong data about the effectiveness of such drugs in the management of COVID-19.

In the present study, 20% of the patients were transferred to PICU. The exact PICU admission rate in children with COVID-19 remains unknown. Götzinger et al. reported an 8% PICU admission rate in their study in Europe [8]. In Spain, only 16% of confirmed cases were admitted to the PICU [27]. In another study in New York City, the rate of PICU admission was 28% in 46 hospitals [22]. Although some of these studies show a higher rate of critical illness than previously reported, detailed clinical characteristics and multicenter longitudinal outcomes were not reported. The difference in results may be attributed to different PICU admission policies. Also, since we are a referral hospital, all critically ill children needing PICU admission are referred and this increases the number and duration of PICU admission and hospitalization.

In this study, IVIG and packed cells were prescribed for 11 and 13 children with PIMS, respectively. High-dose IVIG (2 g/kg) is considered a treatment modality for COVID-19 patients with PIMS and may decrease the risk of coronary artery disease [28]. In Derespina et al.'s study, none of the patients received IVIG or convalescent plasma [21]. Chen et al. analyzed the treatment of 99 Wuhan patients with COVID-19 and found that 27% of these patients had received IVIG [29]. Riollano-Cruz et al. used IVIG in 80% of their cases with PIMS [30]. Its practical application value in the treatment of COVID-19 needs confirmation in future studies. We prescribed IVIG for patients with coronary artery or cardiac involvement before distinguishing between Kawasaki disease or COVID-19-associated PIMS which improved their outcome.

PIMS presented in 13% of our patients, and all of the mortalities occurred after this event. Different studies were alarmed about the severe form of COVID-19 in children which contributed to inflammatory storm and multiorgan failure [6, 24, 30–34].

In this study, 14% of the patients had comorbid diseases and the mortality rate was 4%. The mortality of COVID-19 in children was low in previous studies [12, 17]. The presence of comorbidities is a risk factor for the development of critical illness. In Derespina et al.'s study, 74.3% of the patients admitted to the PICU had at least one comorbidity and 2.9% of the children died [21]. Chao et al. reported one mortality out of 67 patients (1.49%) in their study [22]. Three of our deaths in this study occurred in the first month of the COVID-19 pandemic in other centers, and patients who died had underlying diseases in addition to thrombocytopenia and pulmonary hemorrhage.

This study has the limitations of incomplete medical records and lack of access to chest CT-scan and RT-PCR in some hospitals. Since children with history of contact with confirmed COVID-19 cases and CT findings compatible with COVID-19 were considered a COVID-19 patient, we performed RT-PCR in only 54 cases in which 17 cases were positive. Further studies are needed to better understand underlying pathophysiologies and potential spectrum versus distinctive clinical conditions of the COVID-19 in children.

## 5. Conclusion

The prevalence of COVID-19 in children is lower than adults, and the most severe form of the disease is PIMS. Mortality is low in this age group and usually occurs in patients with underlying disease or morbid obesity. COVID-19 can cause symptoms in children in two stages. In the first week, upper and lower respiratory symptoms can occur which has lower severity and prevalence compared to adults. But after 2-3 weeks following infection, symptoms of MIS-C or multisystem involvement can occur and COVID-19 should be considered. The most common indication for admission is fever, rash, and respiratory problems.

## Figures and Tables

**Table 1:** Demographic data and clinical characteristics of the children with COVID-19 (*N* = 100).

		Frequency
Age^∗^	<1 years^∗∗^	17
	1-4 years	17
	5-11 years	21
	12-18 years	45
Gender	Male	57
	Female	43
Living place	Urban	70
	Rural	30
Admission ward	ICU	20
	Non-ICU	80
Outcome	Improvement	96
	Death	4
PCR (*n* = 54)^∗∗∗^	Positive	17

^∗^Under 5 years: *N* = 34 and 5 years and above: *n* = 66. ^∗∗^Under 28 days: *N* = 3. RT-PCR was performed for 54 cases.

**Table 2:** Therapeutic modalities of admitted children with COVID-19 (*N* = 100).

		Frequency
Measures taken (*N* = 62)	Oxygen with mask	51 (82.26%)
	Oxygen with hood	6 (9.68%)
	Intubation	5 (8.06%)
Measures	IVIG	11
	Packed cell	13
	Albumin	9
	FFP	4
Drug treatment (*N* = 100)	Oseltamivir	15
	Ribavirin	4
	Kaletra	19
	Chloroquine	44
	Tavanex	8
	Vancomycin	33
	Meropenem & imipenem	26
	Ceftriaxone	44
	Azithromycin	19
	Ampicillin	8
	Co-amoxiclav	4

^∗^All 4 reported deaths were intubated.

**Table 3:** The laboratory findings of the patients with COVID-19 at admission time (*n* = 100).

	Mean ± SD (range)		Frequency
WBC	10.78 ± 6.91 (2.90-52.5)	Leukopenia (≤4000/*μ*L)	7
		Leukocytosis (≥5000/*μ*L)	12
Hb	11.35 ± 2.25 (5.60-20.0)	Hb ≥ 10 g/dL	59
PLT	288853.16 ± 163878.62 (3400-949000)	Thrombocytopenia (<150000/*μ*L)	12
		Thrombocytosis (≥450000/*μ*L)	11
ESR	41.04 ± 30.67 (5.0-116.0)	ESR > 30 mm/h	37
CRP	—	CRP+	51
BUN	20.35 ± 38.18 (5.0-335.0)	BUN > 40 mg/dL	3
Cr	0.81 ± 1.06 (0.30-10.0)	Cr > 1.5 mg/dL	2
Na	137.38 ± 4.13 (128.0-145.0)	—	—
K	4.24 ± 0.57 (3.10-6.50)	—	—
Ca	9.48 ± 0.83 (8.0-11.4)	—	—

**Table 4:** The primary symptoms of the children admitted with COVID-19 infection.

Symptoms	Frequency
Fever	81
Chills	25
Anorexia	45
Fatigue & weakness	33
Myalgia	17
Inability eat & speak	11
Loss taste	3
Loss smell	3
Dermatologic: skin rash	14
Respiratory	*N* = 79
Cough	68
Shortness breath	56
Sneezing	19
Sore throat	13
Rhinorrhea	11
Chest pain	9
Tachypnea	61
Cyanosis	3
Grunting	4
Nasal flaring	4
Subcostal intercostal retraction	11
Mottling	2
Crackles fine	18
Crackles coarse	9
Wheezing	9
Sound reduction	4
Gastrointestinal	*N* = 47
Nausea vomiting	39
Diarrhea	21
Abdominal pain	19
Total neurologic	29
Confused	15
Headache	14
Sleepiness	14
Convulsions	3
Urinary	*N* = 2
Oliguria	2

## Data Availability

The trial data used to support the findings of this study are available from the corresponding author upon request.

## Abbreviations

COVID-19: Coronavirus disease 2019
CT: Computed tomography
HIS: Hospital information system
IVIG: Intravenous immunoglobulin
PIMS: Pediatric inflammatory multisystem syndrome
RT-PCR: Reverse transcription-polymerase chain reaction
SARS-CoV-2: Severe acute respiratory syndrome coronavirus 2.

## References

[1] Kamali Aghdam M., Jafari N., Eftekhari K. (2020). Novel coronavirus in a 15-day-old neonate with clinical signs of sepsis, a case report. *Infectious Diseases*.

[2] W-j G., Z-y N., Hu Y. (2020). Clinical characteristics of coronavirus disease 2019 in China. *New England Journal of Medicine*.

[3] Park K. S., Kim Y. H., Yeom H. S. (2020). COVID-19 6-month outbreak infection report as of July 19, 2020, in the Republic of Korea. *Public Health Weekly Report*.

[4] Guarneri C., Rullo E. V., Pavone P. (2021). Silent COVID-19: what your skin can reveal. *The Lancet Infectious Diseases*.

[5] Aygün D., Önal P., Apaydın G., Çokuğraş H. (2021). Coronavirus infections in childhood and vaccine studies. *Turkish Archives of Pediatrics*.

[6] Bahrami A., Vafapour M., Moazzami B., Rezaei N. (2020). Hyperinflammatory shock related to COVID-19 in a patient presenting with multisystem inflammatory syndrome in children: first case from Iran. *Journal of Paediatrics and Child Health*.

[7] Royal College of Paediatrics and Child Health (2020). *Guidance: paediatric multisystem inflammatory syndrome temporally associated with COVID-19*.

[8] Götzinger F., Santiago-García B., Noguera-Julián A. (2020). COVID-19 in children and adolescents in Europe: a multinational, multicentre cohort study. *The Lancet Child & Adolescent Health*.

[9] Önal P., Kılınç A. A., Aygün F., Durak C., Çokuğraş H. (2020). *COVID-19 IN Turkey: a tertiary center experience*.

[10] Zimmermann P., Curtis N. (2020). COVID-19 in children, pregnancy and neonates: a review of epidemiologic and clinical features. *The Pediatric Infectious Disease Journal*.

[11] Hong H., Wang Y., Chung H.-T., Chen C.-J. (2020). Clinical characteristics of novel coronavirus disease 2019 (COVID-19) in newborns, infants and children. *Pediatrics & Neonatology*.

[12] Hoang A., Chorath K., Moreira A. (2020). COVID-19 in 7780 pediatric patients: a systematic review. *EClinicalMedicine*.

[13] Kache S., Chisti M. J., Gumbo F. (2020). COVID-19 PICU guidelines: for high- and limited-resource settings. *Pediatric Research*.

[14] Rahimzadeh G., Ekrami Noghabi M., Kadkhodaei Elyaderani F. (2020). COVID-19 infection in Iranian children: a case series of 9 patients. *Journal of Pediatrics Review*.

[15] Ludvigsson J. F. (2020). Systematic review of COVID-19 in children shows milder cases and a better prognosis than adults. *Acta Paediatrica*.

[16] Qiu H., Wu J., Hong L., Luo Y., Song Q., Chen D. (2020). Clinical and epidemiological features of 36 children with coronavirus disease 2019 (COVID-19) in Zhejiang, China: an observational cohort study. *The Lancet Infectious Diseases*.

[17] Dong Y., Mo X., Hu Y. (2020). Epidemiology of COVID-19 among children in China. *Pediatrics*.

[18] Shekerdemian L. S., Mahmood N. R., Wolfe K. K. (2020). Characteristics and outcomes of children with coronavirus disease 2019 (COVID-19) infection admitted to US and Canadian pediatric intensive care units. *JAMA Pediatrics*.

[19] Parri N., Lenge M., Buonsenso D. (2020). Children with Covid-19 in pediatric emergency departments in Italy. *New England Journal of Medicine*.

[20] Zhang C., Gu J., Chen Q. (2020). Clinical and epidemiological characteristics of pediatric SARS-CoV-2 infections in China: a multicenter case series. *PLoS Medicine*.

[21] Derespina K. R., Kaushik S., Plichta A. (2020). Clinical Manifestations and Outcomes of Critically Ill Children and Adolescents with Coronavirus Disease 2019 in New York City. *The Journal of Pediatrics*.

[22] Chao J. Y., Derespina K. R., Herold B. C. (2020). Clinical characteristics and outcomes of hospitalized and critically ill children and adolescents with coronavirus disease 2019 at a tertiary care medical center in New York City. *The Journal of Pediatrics*.

[23] Navaeifar M. R., Ghazaghi M. P., Shahbaznejad L. (2020). Fever with rash is one of the first presentations of COVID-19 in children: a case report. *International Medical Case Reports Journal*.

[24] Greene A. G., Saleh M., Roseman E., Sinert R. (2020). Toxic shock-like syndrome and COVID-19: multisystem inflammatory syndrome in children (MIS-C). *The American Journal of Emergency Medicine*.

[25] Shahbaznejad L., Navaifar M. R., Abbaskhanian A., Hosseinzadeh F., Rezai M. S. (2020). Clinical characteristics of pediatric inflammatory multisystem syndrome associated with COVID-19. *BMC Pediatrics*.

[26] Whittaker E., Bamford A., Kenny J. (2020). Clinical characteristics of 58 children with a pediatric inflammatory multisystem syndrome temporally associated with SARS-CoV-2. *JAMA*.

[27] Tagarro A., Epalza C., Santos M. (2021). Screening and severity of coronavirus disease 2019 (COVID-19) in children in Madrid, Spain. *JAMA Pediatrics*.

[28] Harahsheh A. S., Dahdah N., Newburger J. W. (2020). Missed or delayed diagnosis of Kawasaki disease during the 2019 novel coronavirus disease (COVID-19) pandemic. *The Journal of Pediatrics*.

[29] Chen N., Zhou M., Dong X. (2020). Epidemiological and clinical characteristics of 99 cases of 2019 novel coronavirus pneumonia in Wuhan, China: a descriptive study. *The Lancet*.

[30] Riollano-Cruz M., Akkoyun E., Briceno-Brito E. (2020). Multisystem inflammatory syndrome in children (MIS-C) related to COVID-19: a New York City experience. *Journal of Medical Virology*.

[31] Belot A., Antona D., Renolleau S. (2020). SARS-CoV-2-related paediatric inflammatory multisystem syndrome, an epidemiological study, France, 1 March to 17 May 2020. *Eurosurveillance*.

[32] Chiotos K., Bassiri H., Behrens E. M. (2020). Multisystem inflammatory syndrome in children during the Coronavirus 2019 pandemic: a case series. *Journal of the Pediatric Infectious Diseases Society*.

[33] Ng K. F., Kothari T., Bandi S. (2020). COVID-19 multisystem inflammatory syndrome in three teenagers with confirmed SARS-CoV-2 infection. *Journal of Medical Virology*.

[34] Riphagen S., Gomez X., Gonzalez-Martinez C., Wilkinson N., Theocharis P. (2020). Hyperinflammatory shock in children during COVID-19 pandemic. *The Lancet*.

